# Words putting pain in motion: the generalization of pain-related fear within an artificial stimulus category

**DOI:** 10.3389/fpsyg.2015.00520

**Published:** 2015-04-30

**Authors:** Marc P. Bennett, Ann Meulders, Frank Baeyens, Johan W. S. Vlaeyen

**Affiliations:** ^1^Centre for Psychology of Learning and Experimental Psychopathology, Faculty of Psychology and Educational Sciences, University of LeuvenLeuven, Belgium; ^2^Center for Excellence on Generalization Research in Health and Psychopathology, Faculty of Psychology and Educational Science, University of LeuvenLeuven, Belgium; ^3^Research Group on Health Psychology, Faculty of Psychology and Educational Sciences, University of LeuvenLeuven, Belgium; ^4^Department of Clinical Psychological Science, Faculty of Psychology and Neuroscience, Maastricht UniversityMaastricht, Netherlands

**Keywords:** symbolic generalization, pain-related fear, chronic pain disorders, fear-avoidance model, acceptance and commitment therapy

## Abstract

Patients with chronic pain are often fearful of movements that never featured in painful episodes. This study examined whether a neutral movement’s conceptual relationship with pain-relevant stimuli could precipitate pain-related fear; a process known as symbolic generalization. As a secondary objective, we also compared experiential and verbal fear learning in the generalization of pain-related fear. We conducted an experimental study with 80 healthy participants who were recruited through an online experimental management system (*M*_age_ = 23.04 years, SD = 6.80 years). First, two artificial categories were established wherein nonsense words and joystick arm movements were equivalent. Using a between-groups design, nonsense words from one category were paired with either an electrocutaneous stimulus (pain-US) or threatening information, while nonsense words from the other category were paired with no pain-US or safety information. During a final testing phase, participants were prompted to perform specific joystick arm movements that were never followed by a pain-US, although they were informed that it could occur. The results showed that movements equivalent to the pain-relevant nonsense words evoked heightened pain-related fear as measured by pain-US expectancy, fear of pain, and unpleasantness ratings. Also, experience with the pain-US evinced stronger acquisition and generalization compared to experience with threatening information. The clinical importance and theoretical implications of these findings are discussed.

## Introduction

Over the past 30 years, health psychologists have discovered that not just the intensity of pain, but the *fear of pain* is associated with functional disability, physical inactivity, and feelings of anxiety and depression in patients with chronic pain disorder (McCracken et al., 1992; Vlaeyen et al., 1995; Asmundson et al., 1999; Crombez et al., 1999; Zale et al., 2013). Prospective studies have shown that fear of pain predicts the development of chronic pain better than other physiological complaints, such as the severity of the original injury (Jensen et al., 1994; Gheldof et al., 2010). Also, psychological treatments that foster adaptive emotional regulation strategies can lead to meaningful reductions in disability, distress and life dissatisfaction even in the absence of pain reduction (Morley et al., 1999; George et al., 2006; Leeuw et al., 2008; Wicksell et al., 2008, 2010). This evidence collectively suggests that the emotional response to pain is a significant clinical issue that deserves attention in both research and therapy.

The *fear-avoidance model of chronic pain* appeals to associative learning processes to describe how fear of pain leads to the functional disabilities experienced by some patients (Vlaeyen and Linton, 2000, 2012). Here, pain is thought of as an unconditioned stimulus (pain-US) that motivates emotional learning. Pain’s impetus comes from its sensory salience and also the catastrophic cognitions that an individual might have about its consequences, e.g., a belief that pain signifies damaged nerves. Neutral bodily movements that have been paired with pain can therefore signal the possibility of more pain or (re)-injury (conditioned stimulus; CS+) and evoke *pain-related fear*. Safety behaviors might then develop in a desperate attempt to reduce pain and avoid (re)-injury, e.g., adopting rigid gait (Volders et al., 2012). Any transient relief is likely to be attributed to these coping strategies and increase the likelihood that they will be employed again. However, safety behaviors are often so pervasive that they disrupt valued activities and this in turn has a deleterious impact on mood and sense of self.

A challenge for the fear-avoidance model has been to understand patients who are fearful of movements that never featured in pain episodes. In these cases, there appears to be a problematic (over)-generalization of fear to innocuous movements. It could be that a neutral movement evokes pain-related fear because it is proprioceptively similar to a conditioned movement; a process known as *stimulus* *generalization* (Meulders and Vlaeyen, 2013). To examine this possibility Meulders et al. (2013) recently used a voluntary joystick arm movement paradigm and paired a painful electrocutaneous stimulus (pain-US) with a specific movement (e.g., moving left; CS+) and did not pair the pain-US with another movement (e.g., moving right; CS-). In a subsequent testing phase without the pain-US, participants were prompted to perform intermediate movements varying in similarity to the conditioned movements. Those that were more similar to the CS+ evoked more pain-related fear than those more similar to the CS- such that a gradient was observed; the more similarity with CS+ the more fear. These findings broadly indicate that proprioceptive similarity can indeed facilitate the spreading of pain-related fear. In real-life, generalization could exacerbate the difficulties of chronic pain patients as an increasing number of movements come to elicit distress and avoidance behavior.

An interesting observation is that fear can spread to previously neutral events even if they are physically dissimilar from a conditioned stimulus. For instance, a conceptual sameness shared between arbitrary events might contribute to the (over)-generalization in learned fear and this has recently been referred to as *category-based* or *symbolic generalization* (see Dymond et al., 2014; Dunsmoor and Murphy, 2015)^1^. For example, Dunsmoor et al. (2012) demonstrated that when members from a specific category (e.g., types of tools) are paired with a pain-US, other members spontaneously produce heightened fear in the absence of the US (also see, Boyle et al., in press). One method to study the symbolic generalization of fear involves the creation of artificial verbal categories with perceptually distinct stimuli, e.g., nonsense words or shapes. This is accomplished using a computer-based, operant learning procedure called a matching-to-sample (MTS) task. A single item (the *sample stimulus*) is presented onscreen for a few seconds and is followed by a set of other items. Participants then select one item from the set. From trial to trial, different sets are shown but there is always one correct item (the *comparison stimulus*): correct choices are reinforced (“Correct” appears onscreen) while incorrect choices are punished (“Wrong” appears onscreen). As such, a number of stimulus relations first are taught using corrective feedback wherein different comparison stimuli are mutually related to a common sample stimulus. In a later phase, the emergence of untrained (or derived) stimulus relations is examined using a similar format but without corrective feedback. This phase examines whether participants can reverse the previously trained stimulus relations: if presented with a comparison stimulus then they might select the appropriate sample stimulus from a set of items (*derived symmetry relations*). It is also examined whether participants can combine the previously trained stimulus relations: if presented with one comparison stimulus then they might select another comparison stimulus from a set of items (*derived equivalence relations*). Overall, physically distinct stimuli become functionally substitutable with one another and, therefore, are said to partake in a *stimulus equivalence category*. This emergent interchangeability between distinct stimuli arguably resembles a conceptual sameness between individual items in a natural language category (see, Sidman, 1971; Hayes et al., 2001; Barnes-Holmes et al., 2005). To study the extension of learned fear through these *de novo* verbal categories, an aversive US is repeatedly paired with one of the comparison stimuli (CS+). As a result, other comparison stimuli typically act as if they too predict threat and evoke fear. In this way fear generalizes to stimuli that are perceptually dissimilar to the CS+ and have not been explicitly related to the CS+ but instead share a rather abstract conceptual similarity (Augustson and Dougher, 1997; Valverde et al., 2009; Dymond et al., 2011; Vervoort et al., 2014).

Very little, if anything at all, is known about the symbolic generalization of pain-related fear. Given that visual stimuli can evoke fear based on their membership in verbal categories, it is conceivable that proprioceptive stimuli during movements could also produce fear in this manner. As a real-world example, *lifting* could be thought of as a verbal category entailing different muscular-skeletal movements, e.g., raising a box with the back or picking up an infant with the arms, as well as different vocalizations and written words, e.g., “lift” or “raise.” Should one member of this category become associated with pain then perhaps pain-related fear could generalize throughout this entire category. For example, a well-intended physiotherapist might advise- “*be cautious while lifting because it could damage the spine.*” Here, the category label, “lifting,” becomes conceptually related to pain-relevant, threat attributes, “damage.” This evaluation might then extend to specific movements in the category and precipitate pain-related fear in the absence of a discrete painful experience. Generally speaking, the realization that pain-related fear can spread in accordance with proprioceptive similarity was an important step in the development of a theoretical account of chronic pain-disorders symptoms. Furthering the scope inquiry to consider the complex verbal similarity movements’ share might contribute to a more complete framework.

The current study sought to examine if pain-related fear can emerge due to symbolic generalization. Using a MTS task, two stimulus equivalence categories were established with nonsense shapes, words and joystick arm movements. First, selecting words or performing a movement in the presence of sample shapes was rewarded. Second, derived symmetry relations between movements (or words) and shapes were tested, as were derived equivalence relations between words and movements. Using a pain-related fear conditioning paradigm, a nonsense word from one stimulus equivalence category was associated with a pain-US (CS+) while a nonsense word from the other stimulus equivalence category was not (CS-). Lastly, participants were prompted to perform movements from both equivalence categories and informed that the pain-US could follow; when in truth it never occurred. It was predicted that participants would report heightened pain-related fear for movements equivalent to the pain-relevant nonsense words. Self-reported measures of pain-related fear, retrospective US expectancy and unpleasantness ratings were administered as proxies of pain-related fear. One could also imagine that participants would be more hesitant to initiate movements that are associated with pain. For that reason, it was predicted that movements equivalent to the CS+ would take longer to initiate than movements equivalent to the CS-.

The fear learning literature clearly indicates that fear could be installed through different pathways (Rachman, 1977; Olsson and Phelps, 2004; Dymond et al., 2012), including directly experienced CS–US pairings (e.g., Grillon and Davis, 1997) and verbal threat information (e.g., Field and Schorah, 2007). As a secondary aim, the present study investigated if verbal information about pain alone could catalyze the generalization of pain-related fear to particular movements. Using a between-groups design, one group experienced the CS+ being directly paired with the pain-US while the CS- was not. In a second group, the CS+ was paired with threatening information (e.g., “painful” and “dangerous”) while the CS- was paired with safety information (e.g., “gentle” and “secure”). We predicted that both groups would show generalization of pain-related fear to the actual, equivalent movements. This could mimic the real-life emergence of pain-related fear due to the conceptual relationships between movements and certain evaluative attributes. For instance, and in real life scenarios, words (e.g., “lifting”) are paired with verbal information (e.g., “is dangerous”) and this can prompt evaluative change in the specific referents (i.e., the musculature involved in lifting; e.g., Muris and Field, 2010).

## Materials and Methods

### Participants

Eighty healthy participants (52 female) were recruited for this study through an online experimental management system (*M*_age_ = 23.04 years, SD = 6.80 years, range = 18–49 years) and paid 8/h remuneration. The ethical committee of the Faculty of Psychology and Educational Sciences of the University of Leuven approved the procedure (S55215). All participants signed an informed consent form. Exclusion criteria were pregnancy, cardio-pulmonary difficulties, diagnosed psychiatric disorders or neurological conditions like epilepsy, and wrist pain. Participants were randomly assigned into one of two groups; the *pain-US* group (*N* = 41, *M*_age_ = 22.95 years, SD = 6.80 years) and the *instructed-US* group (*N* = 39, *M*_age_ = 23.13 years, SD = 5.86 years). Due to an experimenter error, one participant was placed into the wrong experimental condition, hence, the uneven group size. The chosen sample size was based on previous research conducted in our lab (see Vervoort et al., 2014; Bennett et al., in press).

### Apparatus

Experimental sessions were conducted in a sound-attenuated cubicle using a Dell desktop PC (17” monitor with a black background; 1024 × 768 pixels). Stimulus presentations and response recordings were controlled using Affect 4.0 (Spruyt et al., 2010). The *pain-US group* experienced an electrocutaneous stimulus. A commercial constant current stimulator (i.e., DS7A, Digitimer, Welwyn Garden City, England) delivered a 2 ms electrocutaneous stimulation (pain-US) to the wrist of the right hand, via Sensormedics electrodes (8 mm) filled with K-Y gel. An individual pain-US intensity level was decided upon during a pre-experimental calibration procedure (*M*_intensity_ = 16.00 mA; SE = 1.70 mA). The pain-US was reliably aversive as indicated by participants ratings using a pencil and paper Likert scale where 0 = not at all unpleasant and 10 = highly unpleasant (M_unpleasentness_ = 7.33; SE = 0.35). The *instructed-US* group was shown safety and threat information in size 32 white Arial fonts instead of the pain-US. Five threatening terms were used- *injury*, *terrible*, *danger*, *pain,* and *hurt*. Five safety terms were used- *safe*, *secure*, *gentle*, *trust,* and *peace*.

**FIGURE 1:**
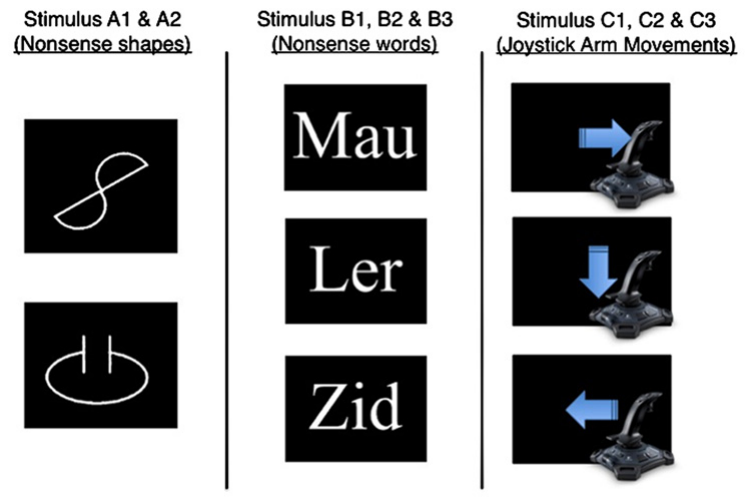
**An overview of the shapes (A1 and A2), nonsense words (B1, B2, and B3) and joystick arm movements (C1, C2, and C3) that were used to establish two separate stimulus equivalence categories (A1 = B1 = C1 and A2 = B2 = C2)**.

**FIGURE 2:**
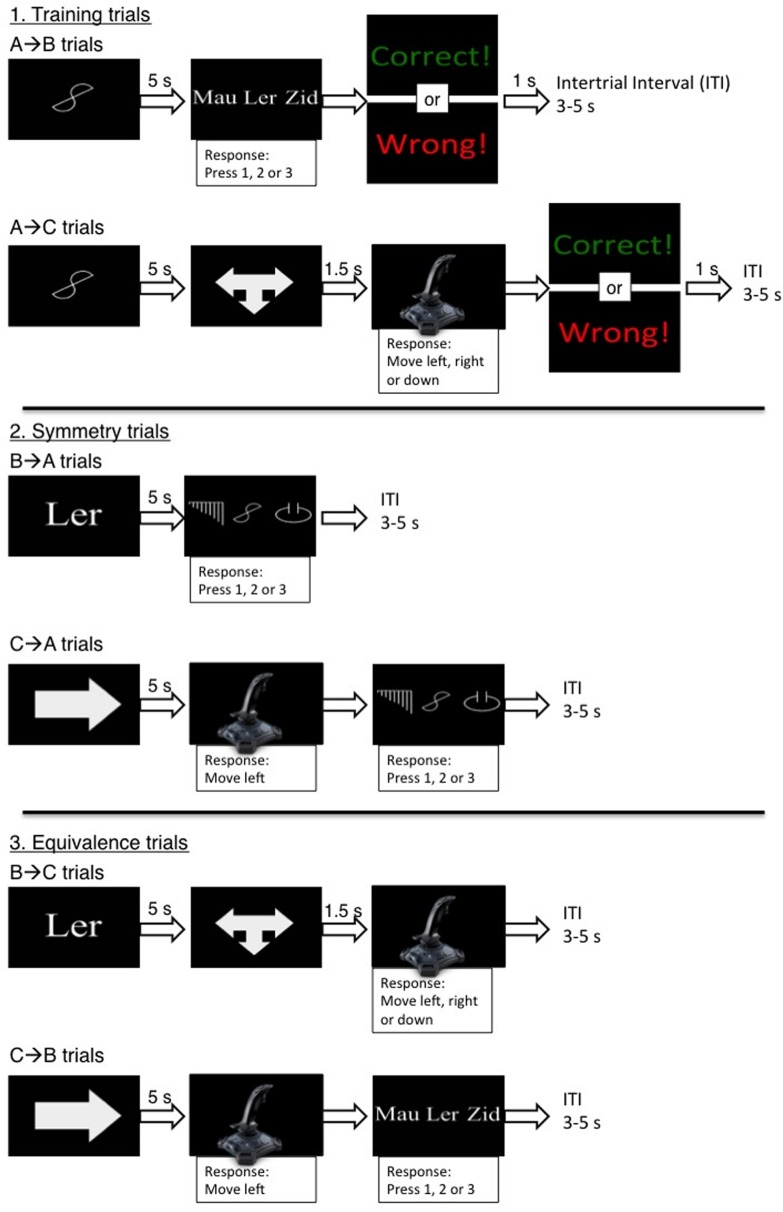
**A schematic overview of the matching-to-sample (MTS) task trials.** The three different panels depict the trial formats for the different MTS phases; (1) training trials, (2) symmetry trials, and (3) equivalence trials. Within each panel is a depiction of the trials types found in a specific phase; training trial phase included [A→B] trials and [A→C] trials; symmetry trials included [B→A] trials and [C→A] trials; and equivalence trials included [B→C] trials and [C→B] trials.

During the MTS task, two nonsense shapes (A1 and A2), 150 × 150 pixels in white font, were used as sample stimuli (see **Figure 1**). Three nonsense three-letter words (B1, B2, and B3) were shown in size 32 white Arial fonts, i.e., “Lur,” “Veg,” and “Mau,” and these acted as comparison stimuli (see **Figure 1**). These words were chosen as previous research has indicated that they are neutral and not associated with a particular evaluative state, prior to conditioning (see Bennett et al., in press). Three arm movements (C1, C2, and C3) were made using a Logitech Attack 3 joystick, i.e., left, right and down, and these acted as comparison stimuli (see **Figure 1**). Joystick was operated by the participants’ right arm and movements were represented as mouse coordinates on the computer screen (the cursor was not visible). A left, right, and downward arm movement was defined by the cursor moving from the middle of the screen, 0 × 0 × 0 × 0 pixels (*top* × *left* × *bottom* × *right*), into a rectangular target region (200 × 200 pixels) positioned at the left side (0 × 284 × 200 × 484 pixels), the right side, (412 × 568 × 612 × 768 pixels), and bottom (824 × 284 × 1024 × 484 pixels) of the screen, respectively. Stimuli were assigned to one of two stimulus equivalence categories; A1 = B1 = C1 and A2 = B2 = C2. During some MTS trials, participants chose to perform one of the three movements and this was cued using a 1.50 s image of three intersecting white arrows pointing left, right and down (50 × 50 pixels); the *comparison-signal* (see **Figure 2**). During other MTS trials, participants were required to perform one specific movement and this was cued using a 5 s image of a white arrow that pointed either left, right, or down (50 × 50 pixels); the *movement-signal* (see **Figure 2**). Participants could only move once the signal was removed from screen and moving too early caused a red X, size 32 font, to appear in the center of the screen. This remained onscreen until the joystick was returned to its resting position, which was defined by a virtual circle located in the center of the screen, 512 × 384 pixels and radius 328 pixels.

### Procedure

#### Pain-US Calibration

Participants confirmed that they did not meet any of the exclusion criteria and signed an informed consent form. They were then brought to the experimental room and a work-up procedure established an intensity of electrocutaneous stimulus for the experiment. The experimenter explained that it was important for the experiment that the electrocutaneous stimulus be uncomfortable and somewhat painful. Two electrodes were placed on the participant’s right wrist, 1.00 cm apart. Starting at 1.00 mA, an electrocutaneous stimulus was delivered with increasing intervals of 1.00 or 2.00 mA until the stimulus was “painful but tolerable.” While progressing upward through these intensities, the experimenter asked the participant to describe aloud the painfulness of the electrocutaneous stimulus, where 0 = feel nothing and 10 = maximum tolerable pain. Once the intensity was selected, participants were asked to rate the unpleasantness of the pain-US using an 11-point Likert scale.

#### Matching-to-Sample Task

##### Pre-training

Six practice trials were completed to familiarize participants with the MTS task and joystick arm movements. Participants were told that the electrocutaneous stimulus would not yet occur. Instructions stated that on some trials a *sample* *stimulus* (A1 or A2) would first appear at the center of the screen and they would then have to choose one of three movements to perform (C1, C2, or C3). Participants were told that the presentation of the *comparison-signal* in the center of the screen would indicate when they were required to choose a movement. Finally, participants were instructed to only perform a movement once the signal terminated and that moving too soon would cause a red X to appear. Over three trials, A1 or A2 randomly appeared at the center of the screen for 5 s. The offset of the sample stimulus was followed by the comparison-signal for 1.50 s in the center of the screen (e.g., **Figure 2**, A→C trials). Over three trials, the experimenter directed the participant to make each movement following the offset of the comparison signal. No feedback was given.

Instructions then stated that other trials would require performing a movement (C1 or C2 or C3) and then selecting 1 of 3 items. Participants were told that the presentation of a *movement-signal* would indicate the specific movement they needed to perform. Participants were again instructed to only make the movements once the signal disappeared otherwise a red X would appear. Over three trials, a movement-signal for C1, C2, or C3 randomly appeared in the center of the screen for 5 s. When the movement-signal terminated, the experimenter directed the participant to make the movement and this resulted in the presentation of B1, B2, and B3 in a line at the center of the screen. The experimenter explained that they could select the stimulus on the left by pressing 1, select the stimulus in the middle by pressing 2, or select the stimulus on the right by pressing 3 (e.g., **Figure 2**, C→B trials). Selecting a stimulus removed all other stimuli and started the next trial. Again, no feedback was given.

##### Trained stimulus relations

Participants were reminded that they should (i) press 1, 2, or 3 to select items, (ii) choose 1 of 3 movements to perform when the comparison-signal appears, and (iii) perform a specific movement when a movement-signal appears. No further instructions were given for the rest of the MTS task. In the first set of trials, A1 and A2 stimuli were *sample stimuli*, and B1, B2, and B3 were *comparison stimuli* (see **Figure 2**, A→B trials). Two trials were presented; [A1→ **B1**, B2, B3] and [A2→ B1, **B2**, B3] (the correct comparison is shown in **bold)**. Here, A1 (or A2) appeared in the center of the screen for 5 s. Its offset was then followed by the presentation of B1, B2, and B3 in a line at the center of the screen (the linear order was randomized). Selecting B1 (or B2) was reinforced by the following feedback, “Correct,” whereas incorrect responses were followed by the following feedback, “Wrong.” Feedback was presented for 1 s and trials were separated by a 3–5 s intertrial interval (ITI). Trials continued until 12 consecutively correct responses were made. In the second set of trials, A1 and A2 were sample stimuli, and C1, C2, and C3 were comparison stimuli (see **Figure 2**, A→C trials). Two trials were presented; [A1→ **C1**, C2, C3] and [A2→ C1, **C2**, C3]. A1 (or A2) appeared in the center of the screen followed 5 s later by the presentation of the comparison-signal for 1.5 s. Following the offset of the comparison-signal, performing C1 (or C2) was reinforced by the following feedback: “Correct,” whereas incorrect movements were followed by the feedback: “Wrong.” The trials continued until 12 consecutively correct movements were made. In a final set of training trials, participants were presented with a mix of all four trial types; [A1→**B1**, B2, B3], [A2→ B1, **B2**, B3], [A1→**C1**, C2, C3], and [A2→ C1, **C2**, C3]. Trials were presented quasi-randomly (with no more than two consecutive presentations of the same type) until 24 consecutively correct responses were made.

##### Derived symmetry relations

Four trials tested if participants would reverse the relation between the sample and comparison stimuli; [B1→**A1**, A2, A3], [B2→ A1, **A2**, A3], [C1→**A1**, A2, A3], and [C2→ A1, **A2**, A3]. These were presented four times each in a block of 16 trials without feedback. On some trials, B1 or B2 appeared in the center of the screen for 5 s followed by A1, A2, and A3 in a line on the center of the screen (see **Figure 2**, B→A trials). On other trials, a movement-signal appeared for 5 s and then participants performed the appropriate arm movement (C1 or C2). Once the movement was complete, A1, A2, and A3 appeared in the center of the screen. Pressing 1, 2, or 3 to select an item caused all stimuli to be removed from the screen (see **Figure 2**, C→A trials).

##### Derived equivalence relations

Four trials were presented to examine the relationship between comparison stimuli; [B1→**C1**, C2, C3], [B2→ C1, **C2**, C3], [C1→**B1**, B2, B3], and [C2→ B1, **B2**, B3]. These were presented four times each in a block of 16 trials, without feedback. On some trials B1 and B2 appeared in the center of the screen for 5 s followed by a 1.5 s comparison-signal. Participants then chose whether to perform C1, C2, or C3 (see **Figure 2**, B→C trials). On other trials, a movement-signal appeared for 5 s and then participants made the appropriate arm movement (C1 or C2). B1, B2, and B3 then appeared in the center of the screen and one of these was selected (see **Figure 2**, C→B trials).

#### Pain-Related Fear Conditioning

For the *pain-US* group, instructions stated that nonsense words would appear in the center of the screen and that the pain-US could follow. B1 was conditioned to predict the pain-US (i.e., B1 was the CS+). B1 was presented four times for 5 s followed by the onset of the 2 ms pain-US. B1 appeared once for 5 s and was not followed by the pain-US. B2 appeared on screen five times and was never followed by the pain-US (i.e., B2 was the CS-). Trials were presented quasi-randomly, with no more than two consecutive trials with the same stimulus, and separated by a 5–9 s ITI. For the *instructed-US* group, instructions stated that extra information would be given about the nonsense word that had been seen. B1 was presented in the center of the screen five times for 5 s and followed by the onset of a 3 s threatening term, i.e., *injury*, *terrible*, *danger*, *pain,* or *hurt*. B2 was also presented five times for 5 s and followed by the onset of 3 s safety term, i.e., *safe*, *secure*, *gentle*, *trust,* or *peace*. Trials were presented quasi-randomly and separated by a 5–9 s ITI. As such, and in both groups, a member of one equivalence category (A1 = B1 = C1) was associated with a pain-US while a member of the other equivalence category (A2 = B2 = C2) was not.

#### Signaled Joystick Arm Movement Task

Instructions stated that participants were now required to make certain arm movements and that the pain-US might follow certain movements. However, at no point in this task was the pain-US presented. Participants were also reminded to wait until the movement-signals disappeared before moving otherwise a red X would appear. Here, movement-signals for C1 or C2 were presented for 5 s after which participants made the appropriate arm movements. C1 and C2 were randomly presented once each in a single block. Overall, four blocks were presented. Trials could only be completed once a movement was performed and were separated by a 5–9 s ITI.

### Outcome Measures

#### Manipulation Checks

Symbolic generalization requires (i) the establishment of a stimulus equivalence category and (ii) a learned fear response. To check for the first criterion, the number of correct responses during the derived symmetry and derived equivalence phases were recorded. Accuracy scores were then calculated for each participant by expressing the total of correct responses as a percentage of the number of trials in each part. An accuracy score greater than 87.50% (14/16 correct responses) was taken to indicate the successful completion of the symmetry and equivalence phases. The mean number of MTS training trials was also calculated and a one-way analysis of variance (ANOVA) was run to examine if the pain-US and instructed-US group differed in the number of MTS training trials. Three one-way ANOVAs were calculated to examine if the pain-US group and instructed-US group differed in performance during (i) MTS training, (ii) symmetry testing, and (iii) equivalence testing.

To check for the second criterion, participants were asked to report the unpleasantness of the CS+ (i.e., B1) and CS- (i.e., B2). This was assessed at the very end of the experimental study. The question “*How unpleasant did you find this word?*” appeared on the top of the screen with B1 or B2 presented in the center of the screen and followed 1.50 s later an 11-point Likert scale, where 0 = not at all, 5 = uncertain, and 10 = highly unpleasant. A repeated measures ANOVA was then calculated to examine the effect of (i) stimulus and (ii) type of US on unpleasantness ratings for CSs. There was 1within-subjects factor (*stimulus*) with two levels; CS+ and CS-. There was also 1 between-subjects factor (*group*) with two levels; pain-US group (directly experienced the pain-US) and the instructed-US group (informed about threat/safety).

#### Symbolic Generalization of Pain-Related Fear

##### Self-report measures

After the signaled joystick arm movement task, participants were informed that they would be asked a series of questions. Each question appeared on the top of the computer screen with the movement-signal for C1 or C2 in the center of the screen. Answers were provided using a mouse-click on an 11-point Likert scale (where 0 = not at all, 5 = uncertain, and 10 = definitely), which was shown at the bottom of the screen. The first two questions measured retrospective pain-US expectancy for C1 and C2; participants were asked, “*How much did you think the electrical stimulation would follow this movement*?” The next two questions measured pain-related fear for C1 and C2; participants were asked, “*How fearful where you while making this movement?*” The final two questions measured the valence for C1 and C2; participants were asked, “*How unpleasant did you find this movement?”*

The mean pain-US expectancy rating, mean pain-related fear rating and mean unpleasantness of movements were calculated for the C1 and C2 movements. A series of mixed repeated measures ANOVAs were then calculated to examine the effect of (i) stimulus and (ii) group on the self-report measures. For each ANOVA there was 1 within-subjects factor (*stimulus*) with two levels; C1 (equivalent to the CS+) and C2 (equivalent to the CS-). There was 1 between-subjects factor (*group*) with two levels; pain-US (experienced pain) and instructed-US (instructed about threat).

##### Reaction time measures

Response latency was recorded for each movement in each block during the signaled joystick arm movement task. This was defined as time between the termination of the movement-signal and the time taken to initiate an arm movement (the joystick deviating from its resting-position). In accordance with the recommendation of Meulders and Vlaeyen (2013), all reaction times shorter than 250 ms and longer than 3000 ms were eliminated. In addition, mean response latency scores for the C1 and C2 were calculated for each participant, and latencies more than 3 SDs from the mean were eliminated (see Meulders and Vlaeyen, 2013). Overall, 2.35% of the overall data set was discarded. A repeated measures ANOVA was calculated to compare the effects of (i) stimulus and (ii) group on the response latency. This model entailed two within-subjects factor; *stimulus*, which had two levels (C1 and C2) and *block*, which had four levels (blocks 1–4). There was 1 between-subjects factor (*group*), which had two levels (pain-US and instructed-US).

## Results

Where Mauchly’s test revealed that sphericity could not be assumed, the Greenhouse–Geisser correction is reported. The alpha-level was set at 0.05 and effect size was calculated using the partial ETA squared ($\eta_{p}^{2}$). Bonferroni corrections were used as the rejection criteria when pairwise comparisons were calculated.

### Matching-to-Sample Task

A mean of 68.47 MTS training trials (SE = 1.56) were required and there was high accuracy of responding (*M* = 88.99%, SE = 0.57%). The one-way ANOVA indicated that the pain-US group required significantly more MTS training trials than the instructed-US, *F*(1,78) = 4.49, *p* = 0.04, $\eta_{p}^{2}$ = 0.04. However, just four outliers in the pain-US group drove this difference. In addition, a one-way ANOVA indicated that the groups did not significantly differ in terms of accuracy during MTS training, *F* < 1.00, *p* = 0.43. More importantly, a high level of accuracy was achieved during the symmetry testing (*M* = 88.75%, SE = 2.25%). One-way ANOVA indicated that the two groups did not significantly differ in terms of their accuracy during symmetry testing, *F* < 1, *p* = 0.33. Finally, a high level of accuracy was achieved during the equivalence testing (*M* = 89.66%, SE = 2.57%) and a one-way ANOVA indicates that the two groups did not differ in their performance, *F* < 1, *p* = 0.93. The accuracy during the symmetry and equivalence testing suggests that stimulus equivalence categories were reliably established. Therefore, the criterion of the first manipulation check was met.

### Unpleasantness of the Original CSs

**FIGURE 3:**
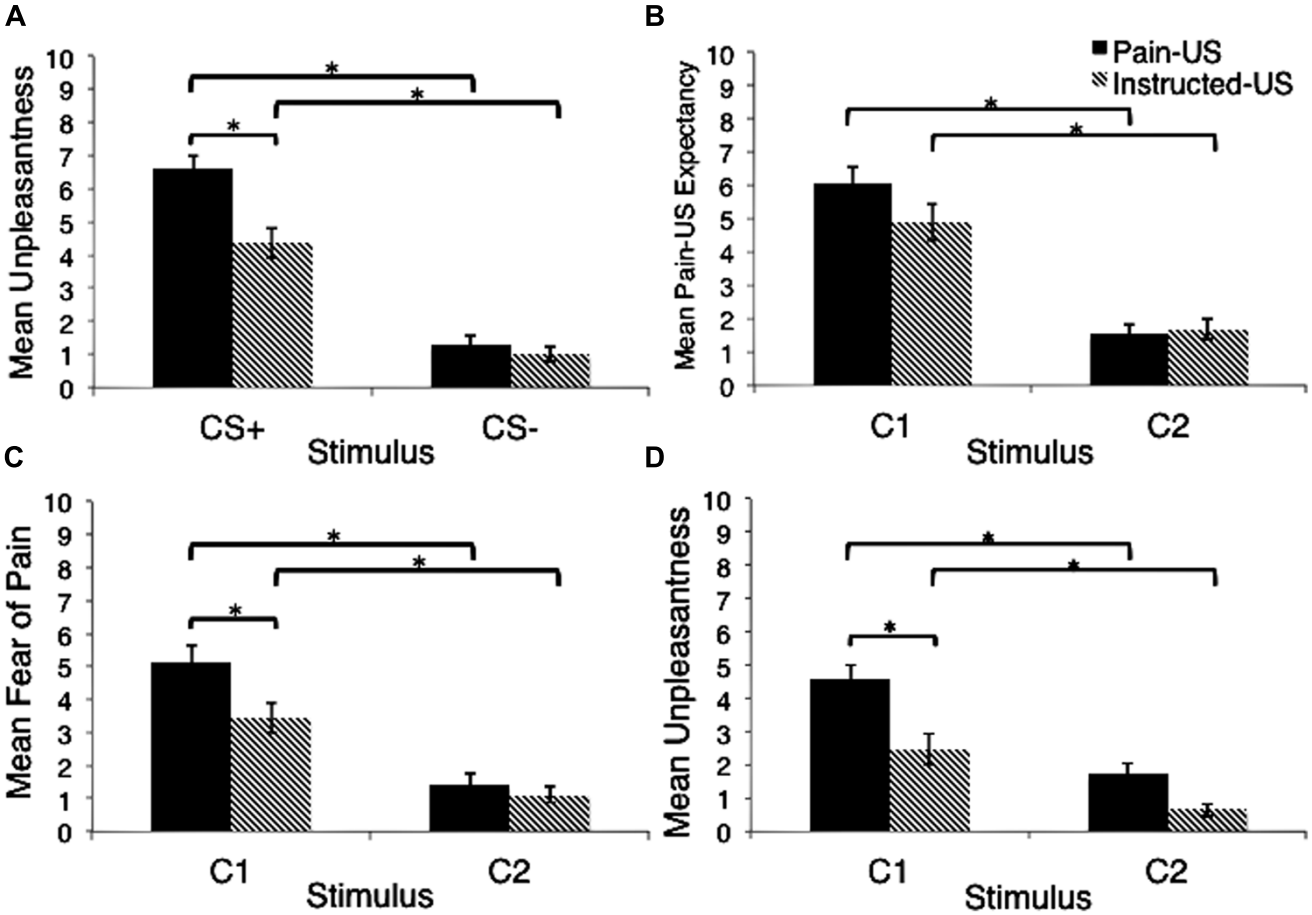
**(A)** Mean unpleasantness ratings for the original CSs. This was a manipulation check to ensure complete conditioning. **(B)** Mean pain-US expectancy ratings for movements equivalent to the original CSs. **(C)** Mean fear of pain ratings for movements equivalent to the original CSs. **(D)** Mean unpleasantness ratings for movements equivalent to the original CSs. Error bars represent standard error. ^∗^*p* < 0.05.

The 2 (stimulus) × 2 (group) repeated measures ANOVA revealed a main effect of stimulus, *F*(1,78) = 148.22, *p* < 0.001, $\eta_{p}^{2}$ = 0.66 (see **Figure 3A**). The CS+ was rated as more unpleasant than the CS- for the pain-US group, *t*(40) = 9.82, *p* < 0.001, *d* = 5.34, and the instructed-US group, *t*(38) = 7.33, *p* < 0.001, *d* = 3.36 (see **Figure 3A**). This suggests that conditioning was complete. The criterion of the second manipulation check was therefore met. Interestingly, a main effect of group was also observed, *F*(1,78) = 14.17, *p* < 0.001, $\eta_{p}^{2}$= 0.15, as was a significant interaction between stimulus and group, *F*(1,78) = 7.69, *p <*0.01, $\eta_{p}^{2}$ = 0.09. The CS+ was rated as more unpleasant in the pain-US group than in the instructed-US, *t*(75) = 3.86, *p* < 0.001, *d* = 2.27. This suggests that the threat value of the CS was higher when paired with the actual US as opposed to threatening information. On the other hand, the two groups did not significantly differ in terms of CS- unpleasantness ratings, *t*(78) = 0.78, *p* = 0.44.

### Pain-US Expectancy

A 2 (stimulus) × 2 (group) repeated measures ANOVA indicated a main effect of stimulus, *F*(1,77) = 94.10, *p* < 0.001, $\eta_{p}^{2}$ = 0.55 (see **Figure 3B**). In line with our predictions, movements equivalent to the CS+ prompted higher pain-US expectancy than movements equivalent to the CS- in both the pain-US group, *t*(40) = 7.91, *p* < 0.001, *d* = 4.53, and the instructed-US, *t*(37) = 5.82, *p* < 0.001, *d* = 3.21 (see **Figure 3B**). There was no main effect of group, *F* = 1.20, *p* = 0.27, nor was there an interaction between group and stimulus, *F* = 2.76, *p* = 0.10. This indicates that the groups did not differ in their expectancy ratings for movements equivalent to the CS+, *t*(77) = 1.60, *p* = 0.12, and movements equivalent to the CS-, *t*(77) = 0.33, *p* = 0.74.

### Fear of Pain

A 2 (stimulus) × 2 (group) ANOVA indicated a main effect of stimulus on self-reported fear of pain, *F*(1,77) = 70.75, *p* < 0.001, $\eta_{p}^{2}$ = 0.48 (see **Figure 3C**). As predicted, movements equivalent to the CS+ evoked higher pain-related fear ratings than movements equivalent to the CS- in the pain-US group, *t*(40) = 6.89, *p* < 0.001, *d* = 3.71, and the instructed-US, *t*(37) = 4.98, *p* < 0.001, *d* = 2.32. Interestingly, a main effect of group was also observed, *F*(1,77) = 5.98, *p* = 0.01, $\eta_{p}^{2}$ = 0.07, and the interaction between group and stimulus was nearing significance, *F*(1,77) = 3.78, *p* = 0.056, $\eta_{p}^{2}$ = 0.05. The pain-US group reported significantly more pain-related fear in response to movements equivalent to the CS+ than the instructed-US group, *t*(77) = 2.54, *p =*0.01, *d* = 1.73. On the other hand, the two groups did not differ in pain-related fear in response to movements equivalent to the CS-, *t*(73) = 0.88, *p* = 0.38.

### Unpleasantness of the Movements

A 2 (stimulus) × 2 (group) repeated measures ANOVA indicated a main effect of stimulus on the self-reported unpleasantness of movements, *F*(1,78) = 40.68, *p* < 0.001, $\eta_{p}^{2}$ = 0.34 (see **Figure 3D**). As predicted, movements equivalent to the CS+ were rated as more unpleasant than movements equivalent to the CS- in both the pain-US group, *t*(40) = 5.19, *p* < 0.001, *d* = 2.83, and the instructed-US group, *t*(38) = 3.79, *p* < 0.01, *d* = 1.61. A main effect of group was also observed, *F*(1,78) = 13.31, *p* < 0.001, $\eta_{p}^{2}$ = 0.15, but there was no significant interaction effect, *F* = 3.04, *p* = 0.09. Interestingly, the pain-US group rated the CS+ equivalent movements as significantly more unpleasant then the instructed-US, *t*(78) = 3.26, *p* < 0.01, *d* = 2.07. Finally, and using Bonferroni’s corrected alpha level (α = 0.01), there was no difference in how the two groups rated the unpleasantness of the CS- equivalent movements, *t*(62) = 2.18, *p* = 0.03.

### Response Latency

**FIGURE 4:**
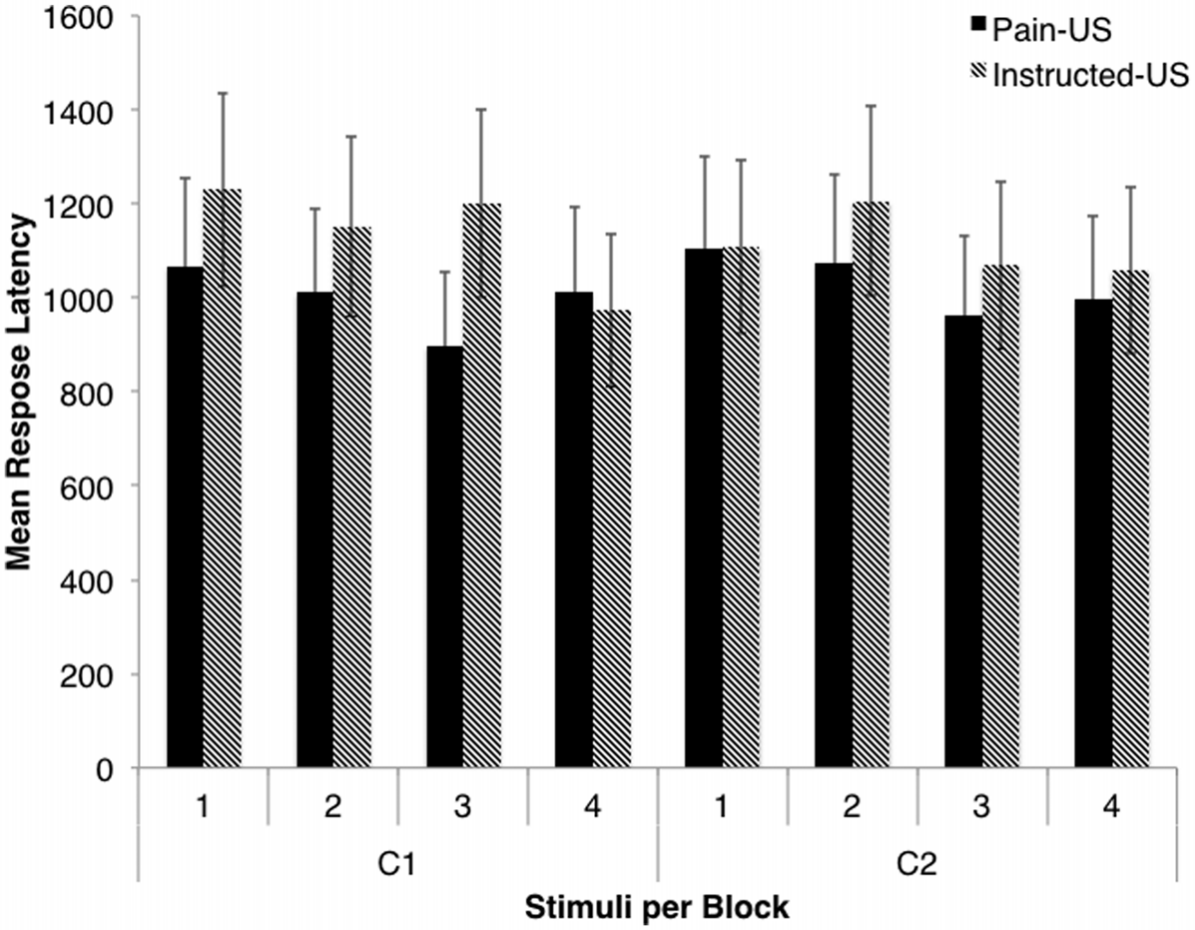
**Response latency as measured during the signaled joystick movement task: the mean response latency shown per block for each stimulus, and for both the pain-US group and instructed US group.** Error bars represent standard error.

A 2 (stimulus) × 2 (group) × 4 (block) ANOVA indicated no main effect of stimulus, *F* < 1, *p* = 0.87 (see **Figure 4**). There was also no main effect of group, *F* = 1.98, *p* = 0.16. There was, however, main effect of block, *F*(3,198) = 5.60, *p* < 0.001, $\eta_{p}^{2}$ = 0.08. Pairwise comparisons indicated that the mean response latency during the first block were significantly longer than those in the third block, *d* = 95.11, SE = 30.91, *p* = 0.02, and fourth block, *d* = 117.22, SE = 36.10, *p* = 0.01. Also, the mean response latency during the second block were significantly longer than those in the fourth block, *d* = 100.47, SE = 34.79, *p* = 0.03. This suggests that participants performed the specific movements quicker as the signaled joystick arm movement task progressed.

## Discussion

Previous research has clearly shown that a conceptual sameness between individual events can facilitate the generalization of learned fear; this has been termed symbolic generalization (Dymond et al., 2011, 2014). The present study investigated if movements could come to specifically evoke pain-related fear in this manner. The results demonstrated that pain-related fear spread from conditioned nonsense word (CS) to joystick arm movements from within the same stimulus equivalence category. In accordance with our predictions, movements from the pain-relevant stimulus equivalence category spontaneously prompted higher pain-US expectancy ratings, fear of pain ratings and unpleasantness ratings than movements from the pain-irrelevant stimulus equivalence category. This finding is particularly interesting given that the movements themselves were never paired with pain-US, nor were the movements in anyway perceptually similar to the nonsense word stimuli that had been associated with pain. It is also interesting given that the movements and nonsense words were never explicitly related to one another. Participants derived the stimulus equivalence category without any corrective feedback during the derived symmetry and equivalence phases. Overall, it appears that movements can become conceptually related to pain-relevant words through a process of stimulus equivalence-based category formation and that this conceptual relation can facilitate the emergence of pain-related fear. To the extent that stimulus equivalence is involved in real-world verbal behavior (for further discussion see, Hayes et al., 2001; Barnes-Holmes et al., 2005; Dymond, 2014), the current study may describe a unique means for movements to evoke pain-related fear in the absence of a pain episode.

The present study also investigated if verbal information about potential harm could also promote the symbolic generalization of pain-related fear. First, nonsense words that were paired with threat information prompted higher unpleasantness ratings than those paired with safety information, suggesting a change in stimulus valence following conditioning. As predicted, movements that were equivalent to the threat-associated nonsense words then evoked higher pain-US expectancy ratings, fear of pain ratings and unpleasantness ratings than movements equivalent to the safety-relevant stimuli. This observation points to the impressive control that verbally relating movements and evaluative terms can have over emotional responding (also see, Blackledge, 2007; McCracken and Morley, 2014; McCracken and Vowles, 2014). Neutral joystick arm movements evoked heightened fear because of a derived equivalence relation with nonsense words, which were themselves paired with threatening information. Overall, this indicates that conceptually linking movement-terms (e.g., “lifting”) to particular evaluative attributes (e.g., “danger” or “safe”) can alter emotional responding to the actual movements.

Response latencies were expected to be longer for movements from the pain-relevant stimulus equivalence category relative to movements from the pain-irrelevant category. This would suggest a hesitation to perform movements associated with pain, and strengthen the claim that pain-related fear and affiliated avoidance behavior generalized through verbal relations. No such difference was observed. However, previous research suggests response latencies may be less sensitive to the generalization of pain-related fear than other fear measurements. Meulders et al. (2013) found that joystick arm movements that were paired with a pain-US elicited an elevated eye-blink startle response and this subsequently generalized to proprioceptively similar movements. On the other hand, longer response latencies were observed for arm movements that were paired with the pain-US but the same was not observed for proprioceptively similar movements. Future research will be required to examine why such an asymmetry is observed across different fear measurements. One commonality between our study and Meulders et al. (2013) was the use of a basic Logitech Attack 3 joystick. Perhaps future research could benefit from the use of more sensitive and informative technologies to measure arm movements (e.g., Houtsma and Van Houten, 2006). On a related note, it will also be important for future research to measure pain-related fear more directly using physiological measures like skin conductance and startle-reflex potentials (e.g., Lissek et al., 2008; Vervoort et al., 2014). This would provide clearer evidence that the conditioning procedure did indeed install fear/safety of the conditioned stimuli. A limitation of the current study is that we rely self-reported stimulus valence for this information.

Importantly, the current findings indicated that direct experience with pain-US is dominant over verbal information in the initial acquisition and subsequent (symbolic) generalization of pain-related fear. Participants who directly experienced the pain-US demonstrated stronger pain-related fear conditioning than those who received verbal threat information. And this heightened acquisition of pain-related fear may have lead to the heightened generalization of pain related fear. That is, movements prompted higher fear of pain and higher unpleasantness ratings when equivalent to CSs that were paired with the pain-US rather than threat information. These results are congruent with recent research found elsewhere. In a within-subjects design, Raes et al. (2014) first paired one stimulus (CS1+) with an electro-cutaneous US and another stimulus (CS2+) with a ‘placeholder’ that represented the US. This placeholder was explained to participants as a way of preventing the delivery of too many shocks so early in the experiment. In a second phase, participants were instructed that both stimuli would be followed by the actual US for real. Prior experience with a CS–US contingency had an additive effect over instructed fear as CS1+ then prompted higher fear ratings than CS2+. It appears that direct experience with CS–US pairings makes a distinct contribution to fear learning over verbal information. Such nuances between different pathways for pain-related fear learning could be consequential in the assessment and treatment of chronic pain. For instance, it may be important to consider whether a patient had any (in)-direct experience with pain to gauge the intensity of pain-related fear and evaluate the risk of generalization. However, we cannot discount the possibility that the between group difference reflects a procedural artifact. During the pain-related fear conditioning, participants were given quite general information about the CS, e.g., MAU→ “hurt” and VEK→ “safe.” Perhaps conditioning effects would be more comparable between the groups if threat information was more specific, e.g., “MAU will be followed by an electric stimulus” (see Raes et al., 2014).

As far as we are aware, no other study has shown that proprioceptive stimuli can partake in stimulus equivalence categories. Although Tierney et al. (1995) designed an innovative MTS task to establish stimulus equivalence categories with haptic stimuli. Three sticks, each of which had a different center of mass, were placed within the grasp of participants but beyond their visual range. Therefore, the sticks could only be discriminated by their haptic properties once they were placed in the participants’ hands. During some training trials, a sample-word was presented and the selection of one stick was reinforced. During other training trials, a stick was placed in the participants’ hands as the sample stimulus and the selection of a different comparison-word was reinforced. Symmetry relations emerged during the testing phase. Participants selected the appropriate previous sample-word when holding a particular stick and, also, selected the appropriate stick when presented with one of the comparison-words. Derived equivalence relations were also observed. Participants selected the appropriate comparison-word stimulus in the presence of one of the sample-words, and vice versa. Tierney et al. (1995) trained the baseline stimulus relations such that the comparison haptic stimulus for one relation was the sample stimulus for the next relation (a *linear* MTS task). As a result, haptic stimuli could only take part in symmetrical relations with words and not equivalence relations. In our procedure nonsense words and proprioceptive stimuli both served as comparison stimuli to a common sample symbol (a *one-to-many* MTS task). A benefit of our approach is that proprioceptive stimuli could be observed to participate in both (i) derived symmetry with the sample symbol and (ii) derived equivalence relations with the nonsense words.

A key finding in the current study is that verbally categorizing movements with pain-relevant words (through stimulus equivalence learning) can create a potential for unwarranted pain-related fear. It is worth mentioning that a very similar, if not an identical, mechanism is supposed by some to be at the core of human psychopathology. *Acceptance and Commitment Therapy* (ACT; Hayes et al., 1999) is a relatively recent addition to the behavior and cognitive therapies, and has been found to significantly improve emotional, social and physical functioning in chronic pain patients (e.g., McCracken and Vowles, 2007, 2008; Vowles and McCracken, 2008; McCracken and Velleman, 2010). A central assertion in ACT is that humans readily infer verbal rules or relationships and this often becomes a problematic source of behavioral control that dominates over actual experiences; this is referred to as *cognitive fusion* (see Vilardaga et al., 2009; Hayes et al., 2013). As a simple example, individuals with chronic pain might conceptualize certain movements as ‘pain-relevant’ and ‘disabling’ and reify this rule, despite the fact these movements might have never causally featured in a pain episode. Cognitive fusion is often described as a therapeutic construct that speculated to be based on fundamental learning processes such as symmetry and equivalence relations, symbolic generalization as well as Pavlovian and operant conditioning (Hayes et al., 1999, 2013). However, a drawback of this novel approach is a paucity of research that clearly describes how learning processes might relate to the components of ACT, like cognitive fusion (see Arch and Craske, 2008; Dymond et al., 2013; Vlaeyen, 2014). In the context of the current study, we demonstrated that emotional response to physical pain can indeed be influenced by verbal relations; this experimental model might elaborate on the learning mechanisms underlining cognitive fusion in chronic pain disorders. Particular arm movements, which were never before painful, controlled pain-related fear because of their derived equivalence to words that were associated with physical pain (also see, Barnes-Holmes et al., 2004; Blackledge, 2007; Dymond and Roche, 2009). This represents a first step in our research unit to investigate the role of verbal categories in the generalization of pain-related fear and chronic pain disorders. In future, it will be important for us to further explore the core learning processes underlining ACT.

## Conclusion

The present study investigated whether joystick arm movements could evoke pain-related fear due to their participation in a *de novo* verbal category. An artificial stimulus equivalence category was established in which nonsense words and joystick arm movements were equivalent. When nonsense words were associated with pain, joystick arm movements from within the same stimulus equivalence category spontaneously elicited pain-related fear. This highlights a unique pathway for the emergence of pain-related fear in the absence of a discrete pain episode. The present study also employed a between-groups design in which words were associated with pain through direct pairing with the pain-US or through verbal information about threat. While both pathways excited the symbolic generalization of pain-related fear, direct experience with the pain-US had a stronger effect. This may be valuable information when considering the etiology of pain-related fear in chronic pain disorders. Finally, and from a broad clinical perspective, we imagine that this experimental study may speak to the learning mechanisms underlining cognitive fusion in ACT. When considering these promising first results, we contend that it will be particularly intriguing for future research to further explore the role of complex verbal relations in the acquisition, and possibly even the attenuation, of pain-related fear.

## Conflict of Interest Statement

The authors declare that the research was conducted in the absence of any commercial or financial relationships that could be construed as a potential conflict of interest.

## Footnotes

1 There could be some confusion between ‘*stimulus generalization*’ and the phenomenon of ‘*(over)-generalization.*’ The former is a principle of learning- novel stimuli that are distinct but similar to a conditioned stimulus can evoke a conditioned response (Kalish, 1969; McLaren and Mackintosh, 2002). The latter term has been recently used to describe a clinical phenomenon whereby innocuous stimuli can evoke problematic emotional states and responses even if they never featured in an aversive learning episode (e.g., Lissek et al., 2005, 2008; Lissek and Grillon, 2010; Hermans et al., 2013; Dymond et al., 2014; Dunsmoor and Murphy, 2015). There is an obvious link between the two concepts. The (over)-generalization of emotional responses could be explained in terms of ‘stimulus generalization.’ However, it is important to recognize that emotional responses can still spread, or ‘generalize,’ to novel stimuli even in the absence of any physical overlap. For instance, the (over)-generalization of fear can be a product of shared conceptual meaning and category-membership (Dunsmoor et al., 2012; Boyle et al., in press). (For more information, see Dymond et al., 2014; Dunsmoor and Murphy, 2015; Bennett et al., in press).
